# P-991. Repercussions of the COVID-19 Pandemic on Maternal and Congenital Syphilis in South Brazil: A Time Series Analysis 2010-2022

**DOI:** 10.1093/ofid/ofae631.1181

**Published:** 2025-01-29

**Authors:** Fernando Echegaray, Christopher Hernandez, Kavya Sundar, Mary C Cambou, Eddy Segura, Marineide Melo, Breno Santos, Ivana Rosangela Dos Santos Varella, Karin Nielsen-Saines

**Affiliations:** David Geffen School of Medicine, Los Angeles, California; David Geffen School of Medicine, Los Angeles, California; Cooper Medical School of Rowan University, Camden, New Jersey; David Geffen School of Medicine University of California, Los Angeles, Los Angeles, California; UCLA, Los Angeles, California; Hospital Nossa Senhora da Conceicao, Porto Alegre, Rio Grande do Sul, Brazil; Hospital Nossa Senhora da Conceição, Porto Alegre, Rio Grande do Sul, Brazil; Hospital Nossa Senhora da Conceição, Porto Alegre, Rio Grande do Sul, Brazil; David Geffen UCLA School of Medicine, Los Angeles, CA

## Abstract

**Background:**

The global rise in maternal syphilis prevalence and congenital syphilis incidence over the past decade has been concerning. In South Brazil, a promising stabilization in these rates has been undermined since the COVID-19 pandemic began. This study examines the pandemic's impact on the epidemiological trends of maternal and congenital syphilis.

Holt-Winters Forecast of maternal syphilis monthly prevalence rates 2010-2022

**Figure:**
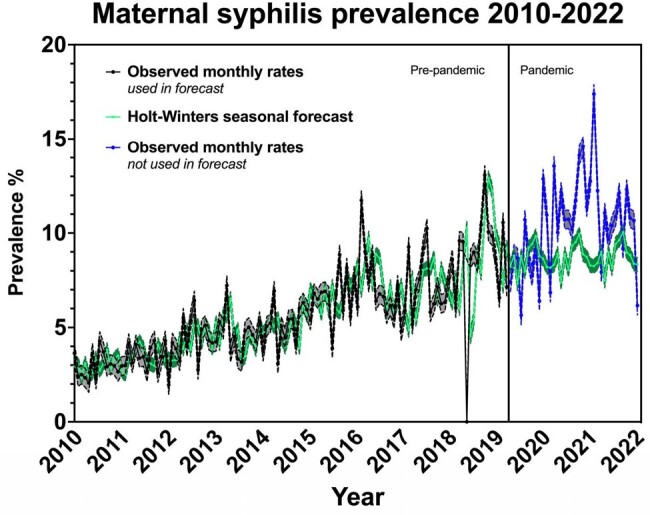

Observed monthly rates used in forecast represented by black dots and connecting black line with gray upper and lower 95% confidence intervals on pre-pandemic panel.

Holt-Winters seasonal monthly forecast shown as lime green dots and connecting lime green line with dark green upper and lower 95% confidence intervals.

Observed monthly rates not used in forecast represented by blue circles and blue connecting line with gray upper and lower 95% confidence intervals on pandemic panel.

**Methods:**

We conducted a retrospective review of hospital records from a South Brazilian tertiary care center, covering the period from January 1, 2010, to December 31, 2022. Assessment of gestational syphilis status was performed using prenatal syphilis records. Maternal syphilis status at delivery and congenital transmission rates were extracted from all recorded deliveries. A Holt-Winters seasonal forecasting model was used to predict maternal syphilis prevalence from pandemic onset, against which we compared actual pandemic period rates. Using data from 2017-2022, a more detailed analysis of total births and corresponding rates of maternal syphilis, congenital transmission, and infant outcomes was performed.

Total deliveries and total maternal syphilis prevalence per year 2017-2022

**Figure:**
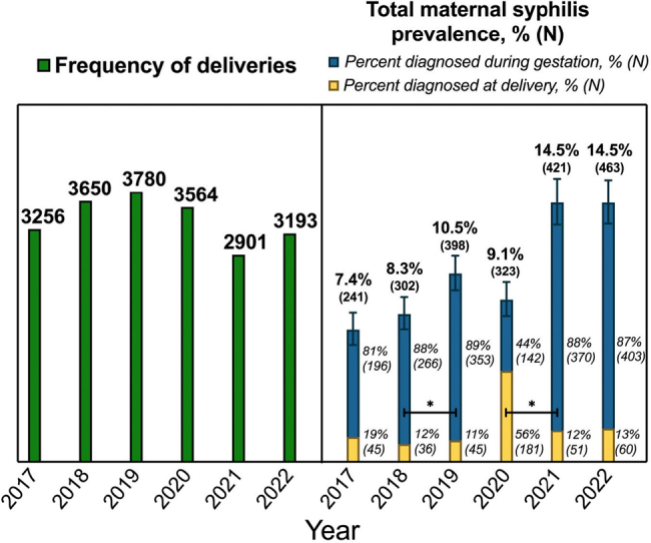

Bar height in green represents total number of births per year in left panel.

Bar height in right panel represents total maternal syphilis prevalence for that year with 95% confidence intervals.

Blue section of bar represents percentage of patients diagnosed with syphilis during prenatal care.

Yellow section of bar represents percentage of patients diagnosed with syphilis at delivery.

* Indicate statistically significant differences (Wilson/Brown p<0.05) between total maternal syphilis rates between years

**Results:**

Projections using 2010-2019 data indicated a potential stabilization in maternal syphilis during the subsequent COVID-19 pandemic years. Contrary to this, the actual rates recorded during 2020-2022 were much higher than those predicted. In 2021 and 2022, forecasting predicted an average yearly rate of 8.7% and 8.8%, while the observed average yearly rate was 12.5% and 10.3%, respectively. Total births throughout 2017-2022 remained stable with a small decline in the pandemic period. With most cases detected at delivery, 2020 saw an artificial decline in total syphilis prevalence at 9.1% that proceeded to increase significantly to 14.5% in both 2021 and 2022. Congenital syphilis transmission initially showed a decrease from 58% to 26% pre-pandemic and rose to 31% in 2022. The incidence of pregnancy terminations and fetal demises in syphilis positive patients declined throughout the period and stabilized at approximately 15 cases per year.

**Figure:**
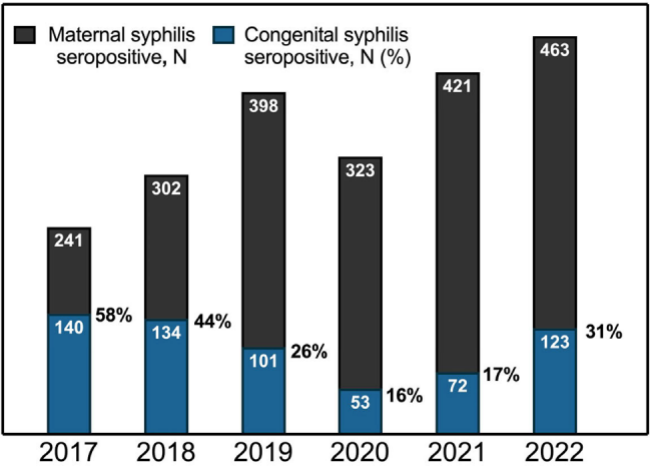

Total black bar height represents total maternal syphilis cases for that year.

Blue section of bar height represents total number of congenital syphilis transmission events for that year.

Percentage next to blue bar height displays congenital syphilis transmission incidence.

**Conclusion:**

The COVID-19 pandemic has been a significant setback in the recent progress made against maternal and congenital syphilis in South Brazil. It is imperative that post-pandemic health strategies prioritize the reinstatement of interventions targeting these infections.

Gestational syphilis infant birth outcomes 2017-2022

**Figure:**
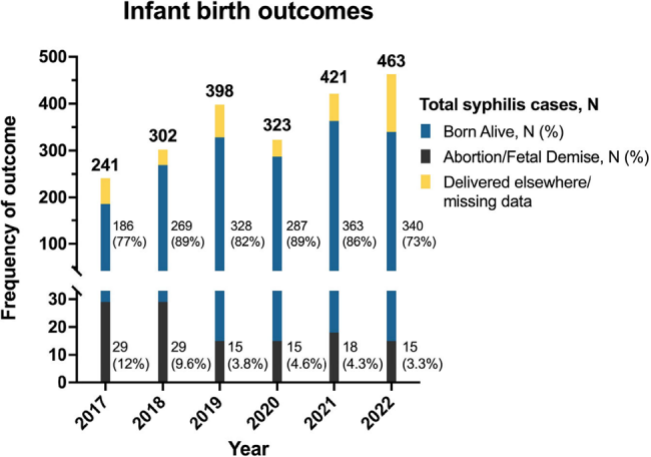

Total bar height represents total maternal syphilis cases for that year.

Blue section of bar height represents total number of infants born alive to syphilis positive patients.

Black section of bar height represents total number of abortions/fetal demises in syphilis positive patients.

Yellow section represents missing data, or patients who delivered elsewhere.

**Disclosures:**

**All Authors**: No reported disclosures

